# *qorA* shapes organ-specific adaptation of ST59-MRSA via balancing immune evasion and metabolic trade-off

**DOI:** 10.1016/j.isci.2026.116272

**Published:** 2026-06-11

**Authors:** Fengning Chen, Yuyao Yin, Si Liu, Hongbin Chen, Yuzhen Wang, Bingqing Li, Yan Jin, Hui Wang

**Affiliations:** 1 Department of Clinical Laboratory, Peking University People’s Hospital, Beijing, China; 2 Department of Clinical Laboratory, Shandong Provincial Hospital Affiliated to Shandong First Medical University, Jinan, China; 3 Department of Pathogen Biology, School of Clinical and Basic Medical Sciences, Shandong First Medical University & Shandong Academy of Medical Sciences, Jinan, China

**Keywords:** Cell biology, Microbiology

## Abstract

Sequence type 59 (ST59) is a predominant methicillin-resistant *Staphylococcus aureus* (MRSA) clone that causes invasive infections in Chinese hospitals. We performed weighted correlation network analysis on clinical MRSA and identified *qorA* as an ST59-specific redox regulator that sustains intracellular homeostasis by promoting NAD^+^-to-NADH conversion. Deletion of *qorA* impaired oxidative stress resistance; reduced the production of SodA, SodM, and staphyloxanthin; triggered metabolic reprogramming; and increased susceptibility to complement C3b deposition and neutrophil-mediated killing; these defects were partially rescued by the antioxidant N-acetylcysteine (NAC). Competitive murine infections demonstrated distinct organ-specific fitness: Δ*qorA* was outcompeted by the wild type in the liver and spleen but exhibited a fitness advantage in the heart, lungs, and kidneys, with comparable bacterial burdens in subcutaneous abscesses. Our findings indicate that *qorA* mediates organ-specific adaptation of ST59-MRSA by balancing immune evasion and metabolic trade-offs, highlighting its potential as a therapeutic target against *S. aureus*.

## Highlights

• QorA maintains NAD^+^/NADH homeostasis in ST59-MRSA
• QorA loss impairs antioxidant defense and immune resistance
• QorA confers liver/spleen benefit but heart/lungs/kidneys cost
• Immune niches drive ST59-MRSA fitness via QorA redox control

## Graphical abstract

**Figure:**
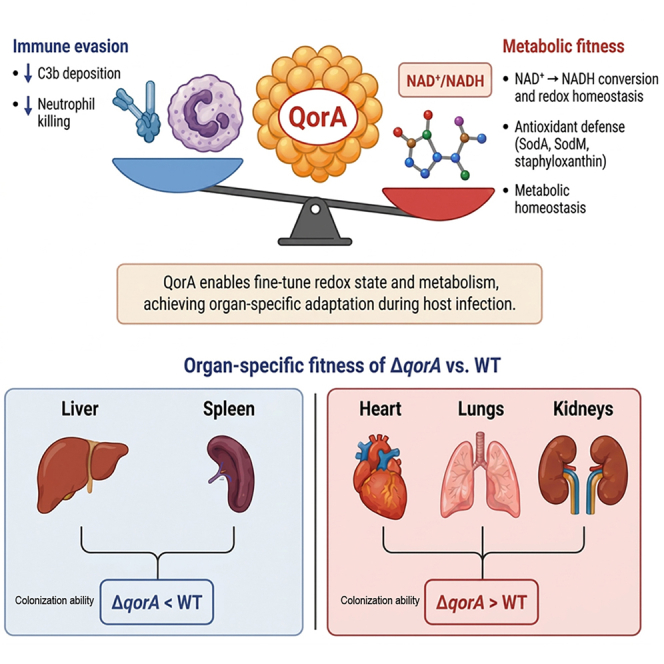

## Introduction

*Staphylococcus aureus* (*S. aureus*) is a major human pathogen capable of causing a variety of life-threatening, invasive diseases and infecting nearly every organ in the human body.^1^^,^^2^ However, *S. aureus* also colonizes the skin and nares in one-third to one-half of the population.^3^^,^^4^ Our group has focused on *S. aureus* clonal changes and first reported the clonal replacement of methicillin-resistant *S. aureus* (MRSA) in China.^5^ ST59 is an epidemic clone in both the Chinese community and hospitals.^6^^,^^7^^,^^8^ Comparative genomic analysis of different clones has been thoroughly studied in our previous research.^8^^,^^9^^,^^10^ This study aimed to identify drivers in the formation of ST59 at the transcriptomic level and investigate the potential role of the key genes that contribute to the pathogenicity and host adaptation of ST59. We employed weighted correlation network analysis (WGCNA) to determine the genetic traits that are specific to the epidemic clone ST59-MRSA. The quinone oxidoreductase *qorA* was identified as a key gene with elevated expression levels in ST59-MRSA compared to other clones. Therefore, we investigated the function of *qorA* in ST59-MRSA and attempted to uncover the underlying mechanisms that impact ST59-MRSA infections.

The ability of *S. aureus* to survive in unique host environments is directly related to its ability to obtain essential nutrients and cope with host.^11^^,^^12^^,^^13^ Therefore, it is vital to understand how pathogens balance self-growth and respond to immune system attacks.^14^^,^^15^ Bacteria must manage reactive oxygen species (ROS) from both internal and external sources. During acute and chronic infections, *S. aureus* must counteract the host’s innate immune system oxidative burst.^16^ Activated macrophages and neutrophils produce large amounts of ROS, as the first line of defense to kill invading pathogens.^17^^,^^18^^,^^19^^,^^20^ Catalase, superoxide dismutase, and glutathione peroxidase are bacterial enzymes that help decrease oxidative stress during aerobic respiration.^21^ However, the effectiveness of these proteins as scavengers of ROS depends on the availability of the reducing substances. Bacteria require available reducing equivalents (NADH and NADPH) to fuel the electron transport chain and supply the reductive power necessary to quell the oxidative potential of ROS.^22^^,^^23^

The innate immune system represents a fast-acting initial line of defense against infection.^12^^,^^19^^,^^24^ To withstand innate defenses, *S. aureus* produces a plethora of virulence factors that enable it to colonize diverse niches and survive insults presented by the immune system, thereby facilitating pathogenesis.^20^^,^^25^^,^^26^ The expression of many of these factors is controlled by transcriptional regulators that respond to the concentrations of primary metabolites,^27^ and it is evident that *S. aureus* can metabolically adapt to improve colonization and overcome challenges imparted by the immune system.^28^^,^^29^ While MRSA possesses a diverse arsenal of toxins, including alpha-toxin, the success of a lineage involves more than just the production of toxins that damage the host. Success is often attributed to the acquisition or loss of genetic elements involved in colonization and niche adaptation, such as the arginine catabolic mobile element, as well as the activity of regulatory systems, which shift metabolism accordingly (e.g., the accessory gene regulator, *agr*).^30^^,^^31^^,^^32^^,^^33^ The importance of microbial metabolic adaptation during infection, coupled with the unclear role of *qorA* in pathogenesis, prompted us to investigate the function of *qorA* in *S. aureus*.^34^^,^^35^^,^^36^

In this study, *qorA* was found to be necessary for maintaining redox balance via NADH synthesis. Deletion of *qorA* disturbed this balance, consequently reducing the levels of antioxidant enzymes and pigments. The Δ*qorA* strain underwent metabolic reprogramming, showing shifts in amino acid and lipid metabolism. Several stress response genes were upregulated after *qorA* deletion, especially the ROS response regulator *comK*, which may facilitate nutrient uptake. Furthermore, we found that the Δ*qorA* strain was more susceptible to neutrophil killing, a defect partially rescued by antioxidant treatment. Most notably, *qorA* conferred a context-dependent fitness advantage: it was crucial for survival in organs such as the liver and spleen, yet appeared to impose a competitive cost in other sites, including the heart, lungs, and kidneys. These findings directly address our initial aim, revealing *qorA* as a key metabolic adaptor that shapes the organ-specific colonization and host adaptation of ST59-MRSA.

## Results

### Different clones exhibit clone-specific clustering in the WGCNA network

**Figure 1:**
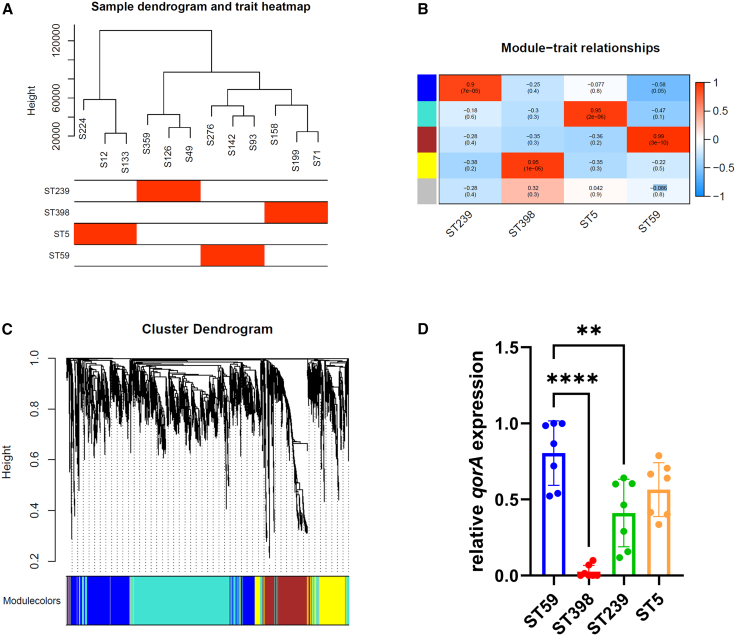
Different clones exhibit clone-specific clustering in the WGCNA network (A) Sample dendrogram and trait heatmap, Sample dendrogram illustrating the hierarchical clustering of samples based on gene expression profiles. The height of the branches indicates the dissimilarity between samples, with closely related samples clustered together. Different clones exhibit clone-specific clustering; ST59-MRSA and ST398-MRSA exhibited the most similarity. (B) Heatmap of the module-trait relationships, showing the correlation between gene modules and different STs. The color scale indicates the strength of the correlation, with red representing positive correlations and blue representing negative correlations. (C) Dendrogram of genes clustered based on their expression similarity. Each branch represents a gene, and the colors indicate different modules identified in the analysis. (D) One-way ANOVA with Tukey’s post hoc test was used to compare differences in *qorA* expression level. Data are shown as mean ± SEM. Each dot represents an MRSA isolate. Each experiment was performed with at least three independent biological replicates and three technical replicates per sample. ∗*p* < 0.05, ∗∗*p* < 0.01, ∗∗∗*p* < 0.001, ∗∗∗∗*p* < 0.0001; NS, not significant (*p* ≥ 0.05).

In our previous study, we identified lineage-specific genes among epidemic *S. aureus* lineages (ST59, ST398, ST239, and ST5) in China.^10^^,^^31^^,^^32^ To investigate clone-specific genes at the transcriptome level, we randomly selected 12 representative strains from four MRSA clones (ST59, ST398, ST239, and ST5). Given that ST59-MRSA is predominant in hospital environments, feature counts were calculated based on a representative ST59-MRSA strain, BS93, and WGCNA analysis was performed on the 12 strains. The analysis revealed distinct clone-specific clustering, with ST59 and ST398 showing the closest relationships (Figure 1A). A total of 2,571 genes were chosen after quality control, and five lineage-related modules were identified (Figure 1B). The brown module was found to be most closely related to ST59-MRSA (Figure 1C; correlation coefficient = 0.99, *p* = 3 × 10^⁻¹⁰^).

After removal of the genomic difference gene and filtering with gene significance > 0.2 and module membership > 0.8, 48 differentially expressed genes (DEGs) were found to contribute to the trait of ST59-MRSA (Table S2). Based on the ranking of count numbers in the top six genes, the first two genes, *ydbp* and *qorA*, were selected for further investigation. While the function of *ydbp* remains to be explored, this study reports the function of *qorA* in ST59-MRSA. To validate the bioinformatics analysis using WGCNA, quantitative real-time PCR was conducted in additional 28 clinical strains, revealing that *qorA* was significantly more highly expressed in ST59-MRSA than in ST398-MRSA and ST239-MRSA (Figure 1D; *p* < 0.0001 and *p* = 0.0025, respectively). A similar trend was observed for ST5-MRSA; however, this finding was not statistically significant.

### *QorA* is essential for redox homeostasis and bacterial fitness

**Figure 2:**
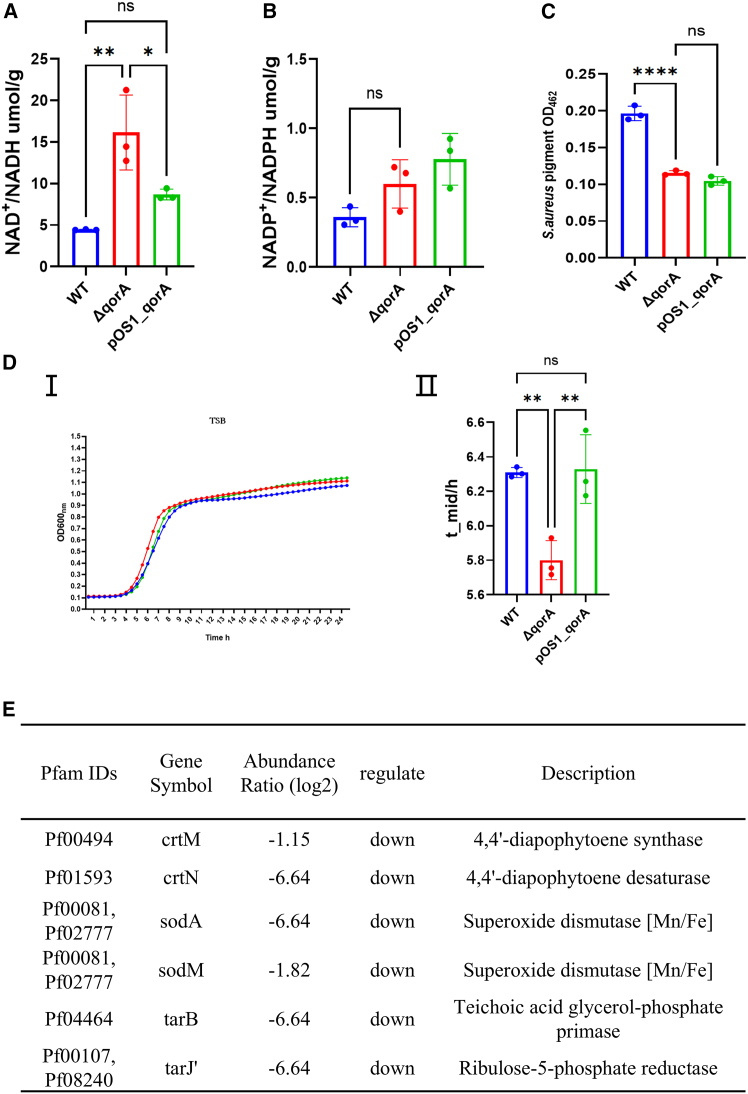
*QorA* maintains redox balance and influences bacterial fitness (A and B) The redox state of WT, Δ*qorA*, and pOS1_*qorA* strains was determined. NAD^+^/NADH and NADP^+^/NADPH ratio were quantified and normalized to bacterial protein concentrations, as assessed by the bicinchoninic acid (BCA) method. (C) Staphyloxanthin production in TSB-grown WT, Δ*qorA*, and pOS1_*qorA* strains. STX production was quantified by absorbance at OD 462 nm. (D) *In vitro* growth curves among the WT, Δ*qorA*, and pOS1_*qorA* strains. Panel I represents cultivation in TSB, and panel II indicates *T_mid* comparison among the WT, Δ*qorA*, and pOS1_*qorA* strains. *T_mid* is the time at which the population density reaches the inflection point. (E) Differentially expressed proteins in the proteome between WT and Δ*qorA* strains. Data are presented as mean ± SEM from at least three independent biological replicates. ∗*p* < 0.05, ∗∗*p* < 0.01, ∗∗∗∗*p* < 0.0001; ns, not significant (*p* ≥ 0.05). Differences were determined by one-way ANOVA with Tukey’s post hoc test for multiple comparisons. Each experiment was repeated independently at least three times.

To investigate the function of *qorA*, we constructed a *qorA*-knockout strain (Δ*qorA*) in a clinical ST59-MRSA strain BS93 (wild-type [WT]) and complemented with the pOS1 plasmid in the knockout strain (pOS1*_qorA*, Table S1). Within the genome of *S. aureus*, *qorA* was annotated as oxidoreductase activity, acting on the CH-CH group of donors and NAD^+^ or NADP^+^ as acceptors. NAD^+^ and NADH are important for cellular energy metabolism, inflammation, and senescence.^37^^,^^38^ NADP^+^ and NADPH are key cofactors in central metabolism and are involved in the tricarboxylic acid cycle, pentose phosphate pathway, and *de novo* synthesis of fatty acids, cholesterol, amino acids, and nucleotides.^39^ Therefore, the NAD^+^/NADH and NADP^+^/NADPH ratios were evaluated; the results showed that *qorA* facilitates the conversion of NAD^+^ to NADH (Figure 2A; *p* = 0.0038), and the complementation strain pOS1*_qorA* restored the ability to transform from NAD^+^ to NADH (*p* = 0.0304). There was no difference in the NADP^+^/NADPH ratio (Figure 2B).

As a reducing substance, the increase in NADH levels contributed to the reduced state of *S. aureus*. Thus, bacteria need to synthesize more oxidizing substances to maintain the internal redox balance, which is in accordance with the phenomenon that staphyloxanthin production was weakened after deletion of *qorA* (Figure 2C; *p* < 0.0001). However, the complemented strain did not reach the WT level, probably because of the influence of the antibiotic resistance plasmids. To elucidate the mechanism of action of *qorA*, unbiased mass spectrometry-based proteomics was used to identify proteins that were differentially expressed upon *qorA* knockout. As the decrease in NADH suggests that the cellular oxidative state was enhanced, superoxide dismutase, SodA, and SodM were downregulated, especially SodA, in the Δ*qorA* strain. Furthermore, we also noticed that the pigment synthases CrtM and CrtN were downregulated in the Δ*qorA* strain (Figure 2E).

The growth abilities of WT, Δ*qorA*, and pOS1*_qorA* were compared, and the results suggested that Δ*qorA* has a decreased *t_mid*, an effect that was fully complemented by the pOS1*_qorA* strain (Figure 2D). *T_mid* is the time at which the population density reaches the inflection point, indicating that *qorA* incurs a fitness cost for *S. aureus.* It produced a far more complex outcome *in vivo*, prompting us to investigate the underlying immunological mechanisms.

### *QorA* deficiency reprograms bacterial metabolism and stress responses

**Figure 3:**
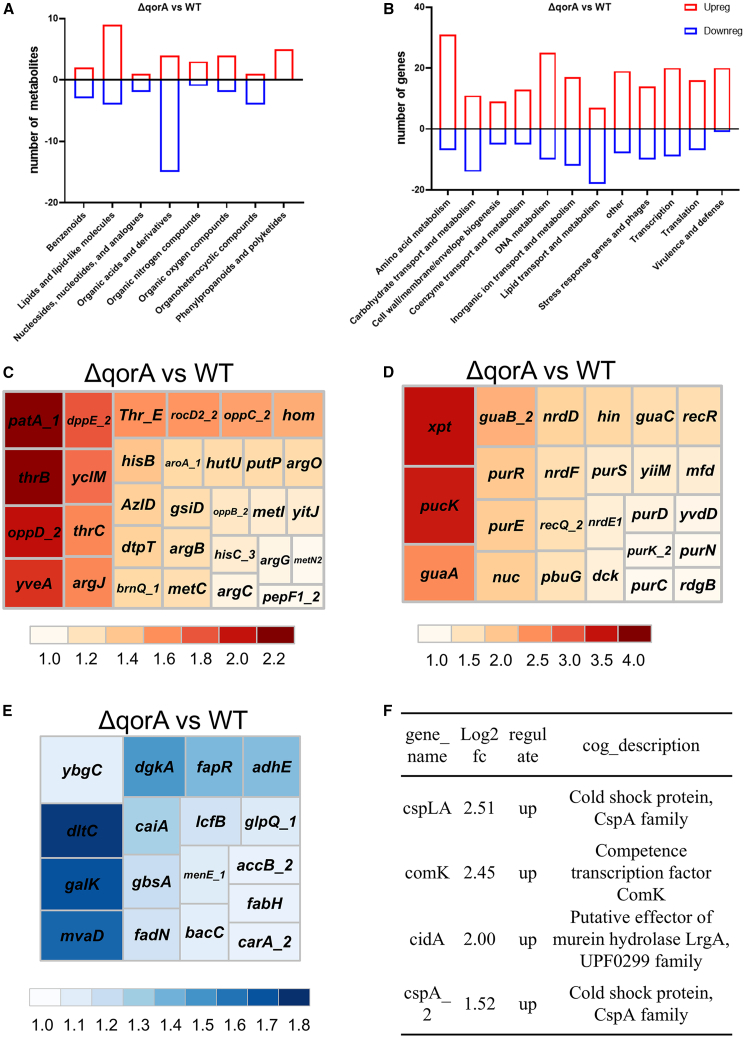
QorA plays a crucial role in modifying the metabolic state of *S. aureus* (A) Classification of metabolites regulated by *qorA* using KEGG_pathway and HMDB. (B) Classification of *qorA*-regulated genes using Cluster of Orthologous Group, Gene Ontology functional categories, and UniProtKB/Swiss-Prot. For each category, columns represent the number of regulated genes from DESeq analyses using a fold-change threshold greater than 2. Pairwise comparisons were made between WT and Δ*qorA* strains. (C and D) Voronoi treemap representing genes downregulated by *qorA* in amino acid metabolism (C) and DNA metabolism (D) Genes in which expression was altered are represented and functionally classified. Each section is labeled with the name of the genes represented. (E) Voronoi treemap representing genes upregulated by *qorA* in the group of lipid transport and metabolism. The Voronoi treemap was generated using the R package “treemap.” (F) Stress response genes that were upregulated in the ΔqorA strain, ranked by fold change.

**Figure 4:**
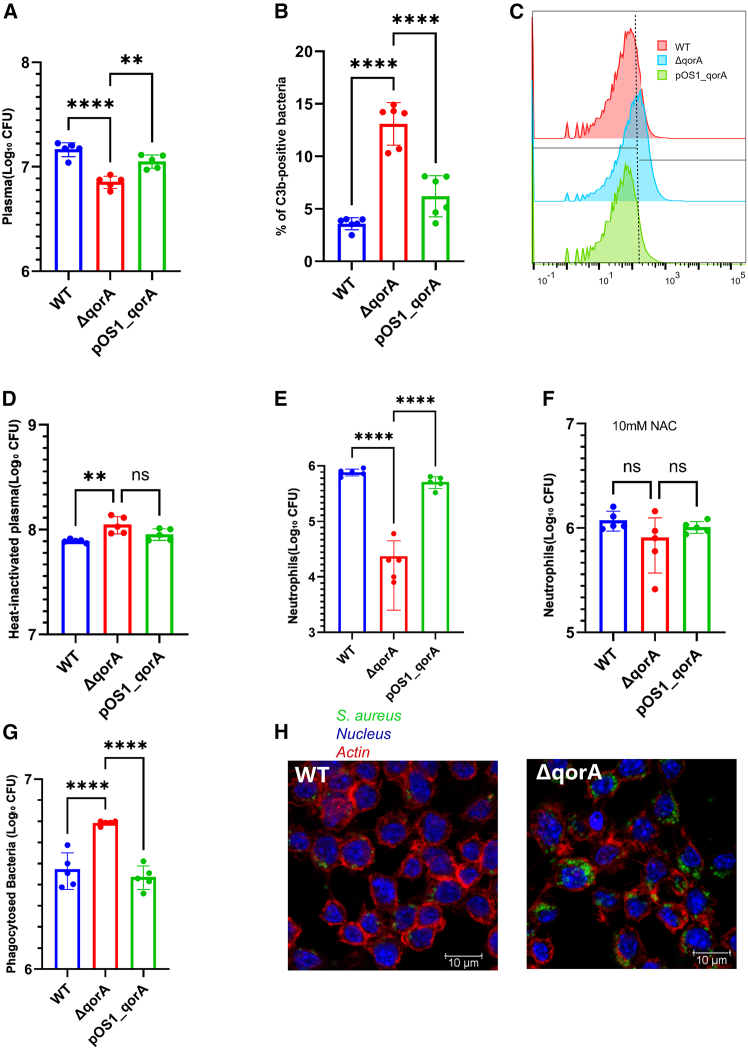
*QorA* deficiency leads to a distinct immune interaction profile driven by redox imbalance (A) Survival of WT, Δ*qorA*, and pOS1*_qorA S. aureus* in human plasma. (B) Percentage of *S. aureus* with surface-bound C3. (C) Quantitative analysis of C3b deposition on *S. aureus*. (D) Survival of WT, Δ*qorA*, and pOS1*_qorA S. aureus* in human heat-killed (55°C for 30 min) plasma. (E and F) Bacterial survival of WT, Δ*qorA*, and the pOS1_*qorA* strains after incubating with freshly isolated neutrophils for 30 min either left untreated or treated with 10 mM N-acetylcysteine (NAC). (G and H) RAW264.7 cells were incubated with WT and Δ*qorA* strain; *S. aureus* was transformed with a GFP plasmid, enabling them to emit green fluorescence; at 30 min after incubation, the cells were examined with confocal microscopy. The red spots indicate the presence of actin, while blue represents the cell nuclei. Scale bars: 10 μm (H). Activated RAW 264.7 macrophages were infected with the WT, Δ*qorA*, or the pOS1_*qorA* strains at a multiplicity of infection (MOI) of 10:1. At 30 min post-infection, extracellular *S. aureus* was killed using gentamicin-supplemented culture medium, and the surviving bacteria were enumerated on TSA plate to calculate the phagocyted rate (G). Data are shown as mean ± SEM. All panels included at least five independent biological replicates, with three technical replicates per group. ∗∗*p* < 0.01, ∗∗∗∗*p* < 0.0001; ns, not significant (*p* ≥ 0.05). Statistical differences were determined by one-way ANOVA with Tukey’s post hoc test for multiple comparisons.

To evaluate the metabolic differences between the WT and Δ*qorA* strains, bacterial untargeted metabolomics was conducted; 31 metabolites were downregulated and 29 metabolites were upregulated in the Δ*qorA* strain (Table S4). Differentially expressed metabolites were divided into eight groups (Figure 3A). Among these, organic acids and their derivatives were predominantly decreased, whereas lipids and lipid-like molecules showed a significant increase in the Δ*qorA* strain. The Δ*qorA* strain’s enhanced survival in heat-killed plasma (Figure 4A) is likely a consequence of its metabolic reprogramming. The global metabolic alterations (Figures 3A and 3B) suggest a compensatory shift in energy metabolism that confers a fitness advantage in this specific condition.

Global gene expression profiling was performed using RNA sequencing (RNA-seq) to identify DEGs between the WT and Δ*qorA* strains. A total of 106 *qorA* upregulated and 202 downregulated genes were identified after excluding genes that encode hypothetical proteins (Table S3), which were classified into different groups using Clusters of Orthologous Groups, Gene Ontology functional categories, and UniProtKB/Swiss-Prot (Figure 3B). Genes involved in amino acid metabolism (*patA, thrB, oppD, yveA, dppE, yclM, thrC*, and *argJ*) were detected among the *qorA* downregulated genes (Figure 3C). Similarly, the DNA metabolism group also contained more *qorA* downregulated genes (Figure 3D). In contrast, *qorA* downregulated the transcription of genes involved in lipid transport and metabolism, such as *dltC*, *galk, mvaD*, and *dgkA* (Figure 3E).

Several stress response genes were upregulated after *qorA* deletion (Figure 3F), such as cold shock protein-encoding genes (*cspA*), *cidA*, and the competence transcription factor *comK*. Low NADH levels can impair energy production and metabolic processes in bacteria, thereby reducing their overall reducing capacities. *comK* expression has been reported to be induced in response to oxidative stress,^40^ which aligns with the elevated NAD^+^/NADH levels observed in the Δ*qorA* strain.

### *QorA* deficiency confers complement hyper-susceptibility and alters phagocyte interaction

We next dissected how the metabolic state of the Δ*qorA* mutant shapes its interactions with key host defenses. The mutant exhibited extreme susceptibility to complement-mediated killing, with its survival rate in active human plasma decreased compared to the WT (Figure 4A; *p* < 0.0001). This survival defect was completely abolished in heat-inactivated plasma (Figure 4D; *p* = 0.0073), pinpointing complement as the decisive factor. The molecular mechanism underlying this phenotype was a significant increase in the deposition of the complement opsonin C3b on the mutant surface, evidenced by both a higher percentage of C3b-positive bacteria (Figure 4B) and a greater median fluorescence intensity (Figure 4C). This directly explains its heightened vulnerability in the bloodstream. Consistent with a role in resisting cellular immunity, the mutant also showed markedly reduced survival upon incubation with purified human neutrophils (Figure 4E; *p* < 0.0001). Critically, treatment with the antioxidant N-acetylcysteine (NAC) partially rescued the mutant’s survival defect within neutrophils (Figure 4F). This rescue experiment directly links these immune vulnerability phenotypes to the core consequence of *qorA* deletion—redox imbalance—establishing a causal relationship.

However, the mutant’s interaction with macrophages revealed a divergent fate. In co-culture assays with macrophages, the Δ*qorA* mutant was phagocytosed significantly more efficiently than the WT strain (Figure 4G; Figure S1; *p* < 0.0001). Confocal microscopy imaging visually confirmed this enhanced uptake, showing a greater association of green fluorescent mutant bacteria with macrophages (Figure 4H). Taken together, these data define a dual immunomodulatory role for *qorA*: it protects the bacterium from both complement and neutrophil killing, while also modulating the efficiency of its uptake upon encounter with macrophages, suggesting an influence on early tissue colonization fate.

### The organ-specific fitness landscape is shaped by local immune microenvironments

**Figure 5:**
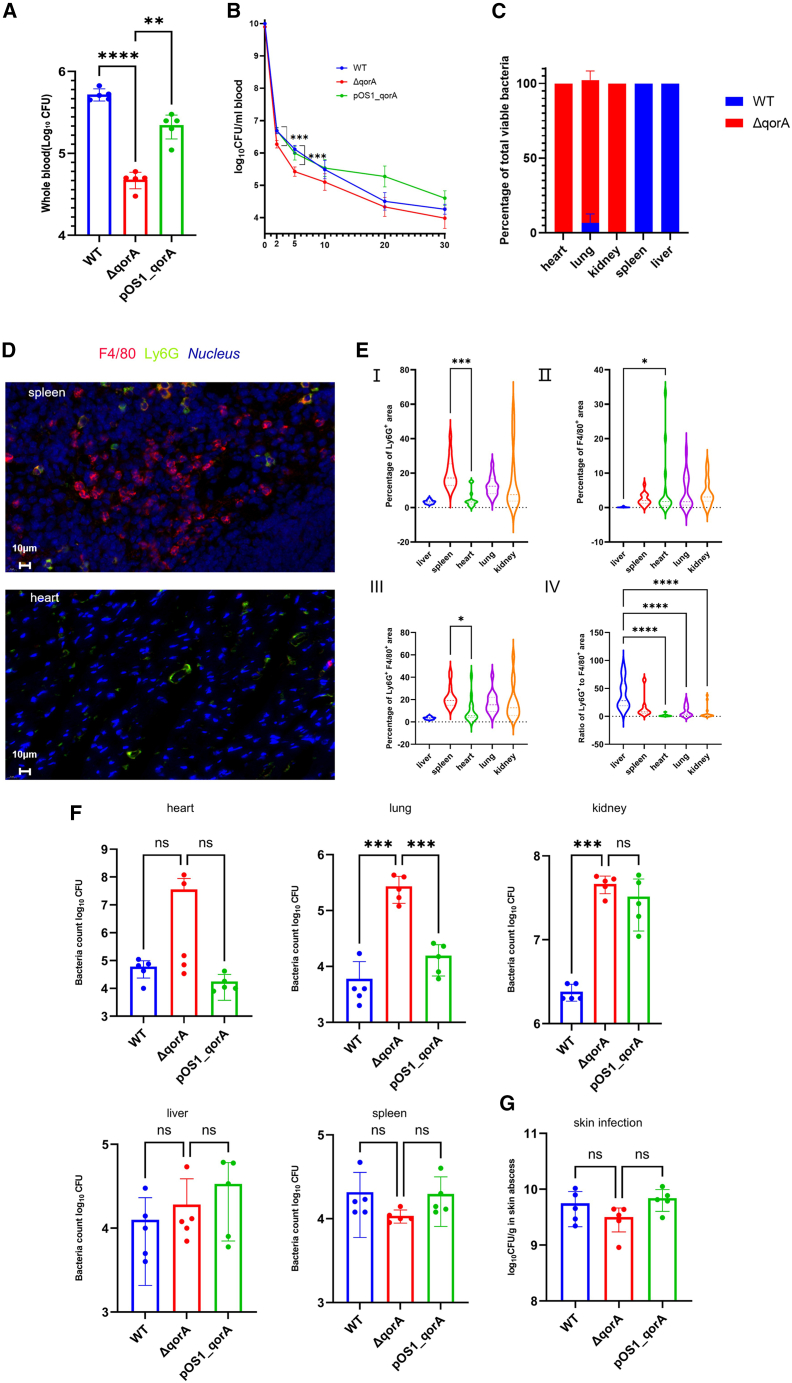
Organ-specific immune microenvironment determines the fitness landscape of the ΔqorA mutant *in vivo* (A) Bacteria survival in whole blood. (B) Murine rapid blood clearance model: mice were infected with WT, Δ*qorA*, and pOS1_*qorA* via tail vein injection. Bacterial enumerations were performed at time zero and at 2, 5, 10, 20, and 30 min after infection. (C) WT and Δ*qorA* strains were mixed at a 1:1 ratio for an *in vivo* competition assay in mice. Following intravenous infection via the tail vein, mouse organs were harvested, and the respective proportions of the WT and Δ*qorA* strains were determined by PCR. (D) Representative immunofluorescence images of infected tissues. Ly6G-positive neutrophils, green fluorescence; F4/80-positive macrophages, red fluorescence; cell nuclei stained with DAPI, blue fluorescence. Scale bar, 10 μm. (E) Quantitative analysis of immune cell infiltration in infected tissues. (Ⅰ) Percentage of Ly6G-positive area, (Ⅱ) percentage of F4/80-positive area, (Ⅲ) comparative analysis of Ly6G-positive and F4/80-positive areas, and (Ⅳ) ratio of Ly6G-positive area to F4/80-positive area. Data are presented as mean ± SEM, *n* = 5 mice per group, 3–5 fields of view per organ. (F) Distribution of bacterial colonies in various mouse organs at 24 h post-bloodstream infection with the WT, Δ*qorA*, and pOS1-*qorA* strains. (G) Bacterial loads in skin abscesses following subcutaneous infection. Each experiment was repeated independently three times with at least five technical replicates per sample. Comparisons were made using one-way ANOVA with Tukey’s post hoc test for multiple comparisons; data are shown as mean ± SEM. ∗*p* < 0.05, ∗∗*p* < 0.01, ∗∗∗*p* < 0.001, ∗∗∗∗*p* < 0.0001; ns, not significant (*p* ≥ 0.05).

To resolve how these contrasting *in vitro* immune interactions translate to the observed organ-specific colonization *in vivo*, we conducted a series of integrated analyses. Consistent with its complement hyper-susceptibility, the Δ*qorA* mutant was rapidly cleared from the bloodstream within minutes of intravenous infection (Figure 5B). Paradoxically and key to our finding, in a direct competitive co-infection assay, the mutant demonstrated a clear fitness advantage over the WT strain in the heart, lungs, and kidneys, but was outcompeted in the liver and spleen (Figure 5C). A 24-h mono-infection model partly confirmed this complex landscape, showing the mutant’s significant advantage in the lungs, and a similar trend in the heart (Figure 5F).

Immunofluorescence analysis of infected tissues elucidated the spatial and mechanistic basis for this organ tropism (Figure 5D; Figure S3). Quantitative assays showed that the proportions of Ly6G^+^ cells and Ly6G^+^ F4/80^+^ double-positive cells were higher in the spleen, with a significant difference compared to the heart but not to the kidneys or lungs; the liver showed no obvious quantitative advantage in these cell populations (Figure 5E). Notably, the liver exhibited a markedly elevated Ly6G^+^/F4/80^+^-positive area ratio, differing significantly from the lungs, heart, and kidneys (all *p* < 0.0001; Figure 5E). Collectively, these organ-specific immune cell composition and ratio biases directly underpin the divergent survival fates of the two strains in distinct host niches. Finally, to determine whether the Δ*qorA* phenotype represented a context-dependent organ-specific adaptation, we employed a subcutaneous skin abscess model. In this distinct anatomical and immunological setting, and in contrast to the dramatic differences observed in systemic organs, the bacterial burdens of the WT and Δ*qorA* strains were not statistically different (Figure 5G). This highlights the precise context-dependent nature of *qorA*’s role in pathogenesis.

## Discussion

ST59 is an epidemic clone in Chinese hospitals, and our group has reported lineage-specific genes of ST59 and attempted to explain the epidemic success of ST59 from a genomic perspective.^9^^,^^31^ In this study, WGCNA was employed to investigate the differences in transcriptomic patterns among four MRSA clones: ST59, ST398, ST239, and ST5. We identified *qorA* as a gene that was specifically highly expressed in ST59 compared to ST239, ST398, and ST5. As WGCNA is a correlation-based method and does not imply causation, we, therefore, constructed *qorA* knockout and complementation strains to investigate its specific function in the ST59 clone. Previous studies have characterized *qorA* as a quinone oxidoreductase in *S. aureus*.^36^ However, the molecular mechanisms governing the metabolic impact and pathogenic potential of *qorA* in ST59-MRSA remain largely unknown.

*S. aureus* demonstrates a remarkable ability to survive and proliferate across various environments during infection, from the skin to deeper tissues and artificial devices. This versatility is indicative of its highly adaptable metabolic capabilities, which allow it to effectively respond to continuously shifting microenvironments.^28^^,^^41^ In this study, we showed that the redox state of *S. aureus* is largely affected by *qorA*, as suggested by the increased NAD^+^/NADH levels in the Δ*qorA* strain (Figure 2A). In contrast to a previous report, which suggested that the activity of *qorA* is similar to that of f-crystallin, which catalyzes the NADPH-dependent one-electron reduction of quinone,^36^ our results showed that *qorA* has no impact on NADP^+^/NADPH transformation but contributes to NADH synthesis in ST59-MRSA. Indeed, the Δ*qorA* strain revealed an impaired reduced state and apparently decreased antioxidant enzyme production, such as superoxide dismutase, SodA, and SodM, and the pigment synthases CrtM and CrtN. Phenotypic experiments on pigment production further confirmed this finding and supported previous observations that golden pigment production is influenced by metabolic processes in *S. aureus*.^42^ However, the *qorA* complement strain did not restore pigment production levels to those of the WT strain, probably because of the effects of the compensatory plasmid pOS1.

The metabolites of organic acids and derivatives were downregulated in the absence of *qorA*, as shown by the metabolome, especially for the amino acid phenylalanine, N-acetylmethionine, and D-proline. One explanation for this phenomenon is that the Δ*qorA* strain tends to utilize amino acids to provide energy and restore redox balance in bacteria. Consequently, in heat-killed human plasma, the Δ*qorA* strain survived better than the WT strain, indicating that *S. aureus* uses another metabolic pathway to acquire essential nutrients for survival. Accordingly, the group of genes involved in amino acid metabolism was upregulated in the Δ*qorA* strain and was mainly related to the synthesis and uptake of amino acids, notably the oligopeptide transport system OPP system genes and arginine biosynthesis genes. In contrast, *qorA* has been shown to deactivate the transcription of genes involved in the synthesis, metabolism, and breakdown of lipids, particularly in fatty acid synthesis and conversion processes. Nevertheless, the levels of lipids and lipid-like molecules, such as fatty acids and glycerolipids, were increased in the absence of *qorA*. Overall, the results revealed that *qorA* altered the metabolic state of *S. aureus*. *QorA* was identified as an RNA-binding metabolic enzyme in *E. coli*,^43^ displaying specific and previously unknown binding to distinct RNAs, such as the interaction of *qorA* with the mRNA of *yffO*, a grounded prophage; however, the biological relevance of this interaction remains elusive. Whether *qorA* plays a role in the RNA-binding function in *S. aureus* requires further investigation.

Several stress response genes were upregulated after *qorA* deletion, such as *cspA*, *cidA*, and *comK*. Among these, we are most interested in *comK*. In *S. aureus*, it has been shown that *comK* is strongly induced during infection and in response to ROS. *comK* upregulates the genes that code for the glucose- and DNA-uptake transport machinery, providing additional nutrients to enhance the fermentation capabilities of bacteria that are unable to respire and a nucleotide source for repair of DNA damage caused by ROS.^40^^,^^44^ In our study, it is reasonable to conclude that the impaired reduced state of *S. aureus* stimulated *comK* expression, thus causing the uptake of amino acids and DNA metabolism. For example, *de novo* guanine biosynthesis is an evolutionarily conserved pathway that creates sufficient nucleotides to support DNA replication, transcription, and translation and is required for *S. aureus* infection *in vivo*.^45^ Deletion of *xpt-pbuX-guaB-guaA* genes resulted in guanine auxotrophy and failure to grow in human serum,^45^ which is consistent with our results. In the DNA metabolism group, the upregulation of the *xpt-guaA-guaB-guaC* genes, along with the induction of *comK* expression due to *qorA* deficiency, may explain why the Δ*qorA* strain reached the logarithmic phase more rapidly under Tryptic Soy Broth (TSB) condition.

Our data point to a significant role for *qorA* in interactions with the host immune system. The Δ*qorA* mutant showed increased susceptibility to complement-mediated killing, associated with greater C3b opsonization, and was cleared more rapidly from the bloodstream (Figures 4A–4C and 5B). It also exhibited reduced survival upon exposure to human neutrophils (Figure 4E). Neutrophils are usually the first cells recruited to the infection site, where they are activated and kill bacteria by an arsenal of antimicrobial processes, such as ROS, antimicrobial granules, and NETosis.^19^^,^^20^^,^^46^ Notably, treatment with the antioxidant NAC partially ameliorated this survival defect (Figure 4F), consistent with the idea that *qorA*’s redox function contributes to neutrophil resistance. In addition, our results support the role of *qorA* in facilitating *S. aureus* survival in human whole blood and a 30-min rapid bloodstream infection clearance model in mice (Figures 5A and 5B). Our study is in agreement with a previous study that observed that *S. aureus* pigment confers resistance to neutrophils and whole-blood killing through its antioxidant activity.^42^ In addition to neutrophils, macrophages are crucial cells in the innate immune defense against infection.^47^ While the mutant was internalized more efficiently by macrophages (Figures 4G and 4H), its subsequent intracellular handling may differ, and the partial rescue by NAC suggests that *qorA* might support survival within macrophages through mechanisms that are not solely dependent on ROS detoxification, possibly involving the altered expression of cell wall-related genes (Figure S2). Genes involved in cell wall, membrane, and envelope biogenesis were differentially expressed in the Δ*qorA* strain compared to those in the WT strain. Collectively, these genes contribute to the synthesis, modification, and integrity of the bacterial cell wall, which can significantly influence the susceptibility to phagocytosis.

The most intriguing finding of this study emerged from the *in vivo* competitive infection assay, which revealed a striking organ-specific fitness landscape that was not apparent in the mono-infection models. In competitive infection, the Δ*qorA* mutant showed a relative advantage in the heart, lungs, and kidneys, but a disadvantage in the liver and spleen (Figure 5C). This pattern could be explained by a context-dependent trade-off. In environments with high phagocytic activity and oxidative stress, such as the liver and spleen, the redox-stabilizing function of *qorA* likely provides a critical survival benefit for the WT strain. Specifically, the liver exhibits an elevated Ly6G^+^/F4/80^+^ ratio and the spleen shows high Ly6G^+^ cell proportions—features consistent with intense innate immune pressure. In contrast, in niches with comparatively lower innate immune pressure, the metabolic burden of maintaining *qorA* may offset its advantage. Notably, our data do not fully exclude the possibility that other uncharacterized factors in the lungs and kidneys may also contribute to the Δ*qorA* advantage, which merits further exploration. This context dependence was organ specific, as no significant difference was found in a skin abscess model (Figure 5G). This phenomenon, where a virulence factor imposes a cost that is only exposed upon direct competition, is a key principle in pathogen evolution. Our findings position *qorA* not merely as a virulence factor but as a central regulator that shapes the ecological adaptation of *S. aureus* within the diverse microenvironments of the host. The incomplete complementation in some organs further suggests that the precise regulation of *qorA* expression is critical for this fine-tuning.

In summary, *qorA* appears to function as a redox modulator in ST59-MRSA, influencing the NAD^+^/NADH balance to support antioxidant defenses and survival against certain immune effectors. Its impact, however, involves a trade-off that seems to depend on the local host microenvironment, thereby helping to shape organ-specific colonization outcomes. This context-dependent role underscores its importance in host adaptation. Further studies are needed to define the signals that regulate *qorA* expression *in vivo* and to assess its potential as a target for therapeutic approaches.

### Limitations of the study

We detected clear shifts in metabolic profiles and gene expression after *qorA* deletion. However, these changes only show a correlative relationship, rather than a confirmed causal connection. We have not yet pinpointed exactly which metabolic pathways or molecular signals directly lead to the distinct fitness differences of the mutant across different organs. More focused mechanistic studies will be necessary to clarify how these alterations directly shape the organ-specific adaptation of ST59-MRSA.

## Resource availability

### Lead contact

Further information and requests for resources and reagents should be directed to and will be fulfilled by the lead contact, Hui Wang (whuibj@163.com; wanghui@pkuph.edu.cn).

### Materials availability

This study did not generate new unique reagents. All plasmids and bacterial strains are available from the lead contact without restrictions.

### Data and code availability

• Data: the RNA-seq, proteomic, and metabolomic data generated in this study have been deposited in the Genome Sequence Archive (GSA) at the National Genomics Data Center, China National Center for Bioinformation/Beijing Institute of Genomics, Chinese Academy of Sciences, under the BioProject accession number PRJCA037513. These datasets are publicly accessible at https://ngdc.cncb.ac.cn/bioproject/browse/PRJCA037513.
• Code: this study did not generate any custom code.
• All other items: any additional information required to reanalyze the data reported in this paper is available from the lead contact upon request.

## Acknowledgments

This work was supported by the 10.13039/501100001809National Natural Science Foundation of China (grant nos. 32141001 and 82202535).

## Author contributions

H.W. conceived and designed the study. F.C., Y.Y., S.L., and Y.W. collected the data and performed experiments. Bioinformatics analyses were performed by F.C. and H.C. F.C. wrote the draft. H.W., B.L., and Y.J. performed the revisions. The authors read and approved the final manuscript.

## Declaration of interests

The authors declare no competing interests.

## Declaration of generative AI and AI-assisted technologies in the writing process

During the preparation of this work, the authors used Gemini (Google) in order to improve the readability and language of the manuscript. After using this tool, the authors reviewed and edited the content as needed and take full responsibility for the content of the published article.

## STAR★Methods

### Key resources table

**Table:** 

REAGENT or RESOURCE	SOURCE	IDENTIFIER
**Antibodies**		

Rabbit anti-Ly6G antibody	Servicebio	Cat#GB11229；RRID:AB_2814689
Rabbit anti-F4/80 antibody	Abcam	Cat#ab6640; RRID: AB_2926938
FITC-conjugated mouse anti-human C3b antibody	BioLegend	Cat#846108; RRID: AB_2813732
Alexa Fluor 488-conjugated secondary antibody	Thermo Fisher Scientific	Cat#A-11001; RRID: AB_2534069
Alexa Fluor 594-conjugated secondary antibody	Thermo Fisher Scientific	Cat#A-11005; RRID: AB_2534073

**Bacterial and virus strains**		

Staphylococcus aureus ST59-MRSA isolate BS93	Clinical isolate, this study	N/A
Staphylococcus aureus BS93 ΔqorA mutant	This study	N/A
Staphylococcus aureus BS93 pOS1_qorA complemented strain	This study	N/A
Staphylococcus aureus RN4220	Laboratory stock	N/A
Escherichia coli *Trans*-T1	TransGen Biotech	Cat#CD201

**Biological samples**		

Human peripheral plasma, neutrophils	Healthy volunteers, this study	N/A
SPF BALB/c mice (female, 6–7 weeks)	Vital River Laboratory Animal Technology	Cat#SCXK-2023-001

**Chemicals, peptides, and recombinant proteins**		

Chloramphenicol	Sigma-Aldrich	Cat#C0378
Ampicillin	Sigma-Aldrich	Cat#A9518
N-acetylcysteine (NAC)	Sigma-Aldrich	Cat#A9165
Tryptic Soy Broth (TSB)	Oxoid	Cat#CM0129
Luria-Bertani (LB) medium	Solarbio	Cat#L1015
Triton X-100	Sigma-Aldrich	Cat#T8787
Paraformaldehyde	Sigma-Aldrich	Cat#P6148

**Critical commercial assays**		

NAD^+^/NADH Assay Kit with WST-8	Beyotime	Cat#S0175
RNeasy Mini Kit	Qiagen	Cat#74104
QuantiTect Reverse Transcription Kit	Qiagen	Cat#RR036A
TB Green Premix Taq™ II	Takara	Cat#RR820A
Ribo-Zero Magnetic Gold Kit	Zhongbei Linge Biotech	Cat#MRZ11124
BCA Protein Assay Kit	Beyotime	Cat#P0012

**Deposited data**		

Transcriptomic, proteomic, metabolomic raw data	Genome Sequence Archive (GSA), NGDC	BioProject: PRJCA037513
Transcriptomic dataset	Genome Sequence Archive (GSA), NGDC	CRA023991
Metabolome data of WT and ΔqorA S. aureus	Genome Sequence Archive (GSA), NGDC	OMIX009546
Proteome data of WT and ΔqorA S. aureus	Genome Sequence Archive (GSA), NGDC	OMIX009579

**Experimental models: Cell lines**		

Mouse RAW 264.7 macrophages	Cell Bank of Chinese Academy of Sciences	RRID: CVCL_0493

**Experimental models: Organisms/strains**		

Mouse: BALB/c	Vital River Laboratory Animal Technology	IMSR_JAX:000651
Staphylococcus aureus: ST59-MRSA BS93	This study	N/A

**Oligonucleotides**		

qorA knockout/complementation/qPCR primers	This study	See Table S1

**Recombinant DNA**		

Plasmid pBTs	Laboratory stock	N/A
Plasmid pBTs_qorA	This study	N/A
Plasmid pOS1	Laboratory stock	N/A
Plasmid pOS1_qorA	This study	N/A

**Software and algorithms**		

GraphPad Prism 8	GraphPad Software	RRID: SCR_002798
WGCNA R package	CRAN	RRID: SCR_003302
FastQC	Babraham Bioinformatics	RRID: SCR_014583
STAR aligner	Cold Spring Harbor Laboratory	RRID: SCR_004463
DESeq2	Bioconductor	RRID: SCR_015687
Proteome Discoverer 2.4	Thermo Fisher Scientific	RRID: SCR_014485
Compound Discoverer 3.1	Thermo Fisher Scientific	N/A
ImageJ	NIH	RRID: SCR_003070
ABI 7500 System Software v2.3	Applied Biosystems	RRID: SCR_003073

**Other**		

NanoDrop 2000 Spectrophotometer	Thermo Fisher Scientific	N/A
Illumina NovaSeq 6000 Sequencer	Illumina	N/A
Thermo Exploris 480 Mass Spectrometer	Thermo Fisher Scientific	N/A
Orbitrap Q Exactive HF-X Mass Spectrometer	Thermo Fisher Scientific	N/A
Olympus FV3000 Confocal Microscope	Olympus	N/A
Flow Cytometer	BD Biosciences	N/A

### Experimental model and study participant details

#### Bacterial strains

*S. aureus* ST59-MRSA clinical isolate BS93 was isolated from a patient with bloodstream infection at Peking University People’s Hospital and identified by MALDI-TOF MS and multilocus sequence typing (MLST). The isogenic Δ*qorA* mutant and complemented strain pOS1_*qorA* were constructed in this study and verified by PCR and Sanger sequencing. All *S. aureus* strains were cultured in Tryptic Soy Broth (TSB, Oxoid) at 37°C with shaking at 220 rpm. Chloramphenicol (10 μg/mL) was added for the mutant and complemented strains when necessary. *Escherichia coli* Trans-T1 was used for plasmid construction and cultured in Luria-Bertani (LB) medium at 37°C with ampicillin (100 μg/mL). *S. aureus* RN4220 was used for plasmid modification and cultured in TSB at 30°C.

#### Cell lines

Murine RAW 264.7 macrophages were obtained from the Cell Bank of the Chinese Academy of Sciences. Cells were cultured in Dulbecco’s Modified Eagle’s Medium (DMEM, Gibco) supplemented with 10% fetal bovine serum (FBS, Gibco) at 37°C in 5% CO_2_. Cells were authenticated by morphological observation and CD11b/F4/80 immunostaining; all cells were tested negative for mycoplasma contamination using a Mycoplasma Detection Kit (Beyotime, Cat# C0296) before use.

#### Biological samples

Human peripheral plasma and neutrophils were obtained from 15 healthy adult volunteers (8 males, 7 females; age range 23–45 years, mean ± SD 31.2 ± 5.6 years) with written informed consent. All samples were processed identically without allocation to experimental groups. The study was not designed to evaluate sex-based differences; therefore, the potential influence of sex on the results cannot be assessed from these data. All protocols involving human specimens were reviewed and approved by the Ethics Committee of Peking University People’s Hospital (Approval No. 2022PHE055).

#### Mice

Specific-pathogen–free (SPF) female BALB/c mice (6–7 weeks old, 18–22 g) were purchased from Vital River Laboratory Animal Technology Co., Ltd. (Certificate No. SCXK-2023-0012). Mice were housed under controlled conditions (22–25°C, 50–60% humidity, 12 h light/dark cycle) with free access to food and water. All animal experiments were conducted in strict accordance with the Laboratory Animal Care and Use Guidelines of the Chinese Association for Laboratory Animal Sciences (CALAS) and the ARRIVE guidelines. The study protocol was reviewed and approved by the Ethics Committee of Peking University People’s Hospital (Approval No. 2022PHE055). All procedures involving animals were performed in compliance with the institutional guidelines for the ethical use of animals in research. Only female mice were used in this study; therefore, sex-specific differences were not investigated, and the findings may not be generalizable to male mice.

### Method details

#### Bacterial strains and growth conditions

Table S1 lists the plasmids and bacterial strains that were used in this study. The strain was isolated from a patient with bloodstream infections in the Clinical Laboratory of Peking University People’ s Hospital. *S. aureus* cells were grown at 37°C, in TSB (Oxoid) supplemented with antibiotics, when necessary. Luria-Bertani (LB) medium (Solarbio) was used to cultivate of *Escherichia coli*. Antibiotics were used at the following concentrations: for *S. aureus*, chloramphenicol (Cm) at 10 μg/ml; for *E. coli*, and ampicillin at 100 μg/ml for *E. coli*.

#### Construction of *qorA* mutant strain

Genetic manipulation of *E. coli* and *S. aureus*. Recombinant plasmids were constructed in *E. coli* trans-T1 cells employing recombinant DNA technology and typical molecular biology. Gram-positive bacteria were used in accordance with a standard procedure to create the genomic DNA of *S. aureus*. Plasmid DNA was extracted using a plasmid purification kit (Promega), according to the manufacturer's instructions. All plasmids transformed into the *S. aureus* strains were firstly introduced into *S. aureus* RN4220 by electroporation, as described previously.^32^

Two fragments that were flanking the upstream and downstream portions of *qorA* were amplified from *S. aureus* BS93 genomic DNA using the primer pairs listed in Table S1. The two PCR products were ligated with the linearized pBTs plasmid using the NEBuilder HIFI DNA Assembly method to generate plasmid pBTs_*qorA* (Table S1), and the two PCR products with a 20-base complementary region to allow ligation, after being converted to RN4220 at 30°C on tryptic soy agar (TSA) containing 10 μg/ml Cm (TSA Cm plates),the plasmid pBTs_*qorA* was converted for modification and then electroporated into the BS93-WT strain. As previously mentioned,^48^ homologous recombination was carried out in the absence of a selection marker. Briefly, individual colonies were streaked onto TSA Cm plates and incubated at 30°C. Single colonies were grown in TSB at 42°C and diluted 1:200 in fresh medium for 2 consecutive days before being diluted and plated on TSA Cm plates to pick for integration into the chromosome. Single colonies were cultured in TSB at 30°C and then diluted in fresh medium for two consecutive days, after which they were further diluted and plated onto TSA plates. Using PCR and sequencing, the *qorA* mutants were checked.

#### Complementation assay

To establish a complementary strain, we amplified the *qorA* operon from the genome of the BS93 strain using the primers listed in Table S1, and reverse amplification was performed using primers to obtain the pOS1 plasmid for ligation with the *qorA* amplification product. The PCR-amplified DNA segments were ligated using the NEBuilder HiFi DNA Assembly system (NEB, E5520S), followed by successive electroporation-mediated transformation of the recombinant plasmid into RN4220 competent cells and subsequently into the *qorA* deletion mutant strain.

#### Weighted gene co-expression network analysis

WGCNA, a methodical approach in the field of biology, is frequently used to describe the patterns of genetic correlations between several samples. We constructed a gene co-expression network for the four epidemic clones ST59, ST398, ST239 and ST5 using the “WGCNA” R package.^49^ The BS93 isolate (ST59) was designated as the reference strain for genomic comparisons. Transcriptomic quantification was subsequently conducted through WGCNA to establish baseline expression profiles. Four distinct characteristics were identified: ST59, ST398, ST239, and ST5. We formed a connection between the modules and their attributes. A gene expression matrix was created, and the top 5000 genes were screened based on their RPKM values. Next, we evaluated the correlation between different modules and various clones, ultimately selecting the most relevant module as the central gene derived from WGCNA. When it came to ST59, the brown module had the best correlation. Key genes were identified after excluding genes with genomic differences, and these were filtered based on their counts, focusing on the top six genes with a GS greater than 0.2 and a MM greater than 0.8.

#### RT-PCR

The RT-PCR analysis was conducted following the previously outlined procedure.^31^ Total RNA was extracted from wild-type *S. aureus* BS93 and Δ*qorA* strains cultured in TSB using the RNeasy Kit (Qiagen). For quantitative PCR (qPCR), MRSA strains were grown to mid-log phase in TSB, and RNA extraction was performed with the RNeasy Mini Kit (Qiagen, Cat. 74104). The first-strand cDNA was synthesized with the QuantiTect Reverse Transcription system (Qiagen, RR036A) in accordance with the manufacturer's specifications, employing 1 μg total RNA as template for reverse transcription. In a 20 μL volumes containing 20 ng cDNA and TB Green Premix Taq™ II on an ABI 7500 Real-Time PCR System (Applied Biosystems, USA), the reaction was carried out.The PCR amplification protocol comprised: (1) initial DNA denaturation (95°C, 30 s); (2) 40 amplification cycles (95°C for 3 s, 60°C for 30 s); and (3) final melt curve analysis. Negative controls without template DNA were run in parallel for all primer pairs (Table S1). Gene expression quantification was performed on the ABI 7500 system (v2.3), with *gyrB* serving as the internal control for ΔCt normalization. Data reflect three independent biological replicates.

#### RNA-seq

Total RNA was extracted from mid-log phase WT and Δ*qorA S. aureus* using the RNeasy Mini Kit (Qiagen). RNA integrity was verified by 1.2% denaturing agarose gel electrophoresis, and purity/concentration were measured using a NanoDrop 2000 (A260/280 > 1.8). Ribosomal RNA was depleted using the Ribo-Zero Magnetic Gold Kit (Zhongbei Linge Biotech, MRZ11124). Strand-specific RNA-seq libraries were constructed using the Illumina TruSeq Stranded mRNA Library Prep Kit. Libraries were quantified and sequenced on an Illumina NovaSeq 6000 platform in 150 bp paired-end mode.Raw reads were quality-filtered using FastQC (v0.11.9) and trimmed with Trimmomatic (v0.39) to remove adapters and low-quality bases (Q < 20). Clean reads were aligned to the *S. aureus* ST59 BS93 reference genome using STAR (v2.7.10a). Transcript abundance was quantified as FPKM using StringTie (v2.2.1). Differential expression analysis was performed using DESeq2 (v1.38.3) with thresholds: |log_2_FC| ≥ 1 and FDR-adjusted *p* value < 0.05. Differentially expressed genes were annotated using COG, GO, and UniProtKB databases.

#### Proteome-wide analysis

Bacterial pellets were lysed in SDT lysis buffer (4% SDS, 100 mM Tris-HCl, 1 mM DTT) and boiled at 95°C for 10 min. Protein concentration was determined by the BCA method. Proteins (10 μg) were reduced, alkylated, and digested with trypsin (1:50 trypsin:protein) at 37°C overnight. Peptides were desalted using C18 StageTips and dried. Nano LC-MS/MS was performed on a Thermo Ultimate 3000 nanoLC system coupled to an Exploris 480 Orbitrap mass spectrometer. Peptides were separated on an Acclaim PepMap RSLC C18 column (75 μm × 250 mm, 2 μm) at 300 nL/min using a 120 min gradient (5–35% acetonitrile with 0.1% formic acid). Full MS spectra (m/z 375–1500) were acquired at 60,000 resolution; MS/MS spectra were acquired in data-dependent mode (top 20 precursors). Raw data were processed using Proteome Discoverer (v2.4). Spectra were searched against a custom *S. aureus* BS93 protein database. Differential proteins were defined with |log2FC| ≥ 1.2 and P value < 0.05.

#### Bacteria LC-MS/MS analysis for untargeted metabolome

Bacterial metabolites were extracted using 80% methanol (v/v) containing internal standards. Samples were centrifuged at 14,000 × g for 15 min at 4°C, and supernatants were analyzed using a Vanquish UHPLC-Orbitrap Q Exactive HF-X system. Separation was performed on a Hypersil Gold C18 column (100 × 2.1 mm, 1.9 μm) with a 17 min gradient. Mobile phases: 5 mM ammonium acetate (pH 9.0) with 0.1% formic acid (A) and methanol (B) for positive/negative ion modes.

Mass spectrometry parameters: spray voltage 3.5 kV, capillary temperature 320°C, full MS resolution 60,000, MS/MS resolution 15,000. Raw data were processed using Compound Discoverer 3.1 for peak alignment, annotation, and quantification. Metabolites were identified using mzCloud, mzVault, and MassList. Differential metabolites were analyzed by PCA and OPLS-DA, with |log2FC| ≥ 1 and VIP > 1 as thresholds. Metabolic pathways were mapped using KEGG and HMDB databases.

#### NAD^+^/NADH assay

Using a NAD^+^/NADH Assay Kit with WST-8 (S0175; Beyotime), the NAD^+^/NADH ratio was determined. The total amounts of NAD^+^ and NADH were expressed with respect to bacterial protein concentration using the BCA method. One-way ANOVA was used to determine the statistical significance, and Tukey's multiple comparison test was used.

#### Plasma survival assay

Bacterial cultures were maintained in TSB at 37°C for 5 hours until achieving logarithmic-phase growth (OD600 = 0.6). Subsequently, 10ˆ8 cells were suspended in 200 μL phenol red-free RPMI 1640 medium and exposed to 30 μL human plasma (heat-inactivated at 55°C for 30 min where specified) during a 37°C incubation period of 30 minutes. Post-treatment, bacterial pellets were collected via centrifugation (12,000 × g, 5 min) and rinsed twice with phosphate-buffered saline (PBS). Viability assessment was performed by plating serial dilutions on TSB agar for quantitative analysis of colony-forming units (CFUs) after 24-hour incubation at 37°C. This assay was performed with 5 independent biological replicates and 3 technical replicates per group; investigators were blinded to group allocation during CFU counting.

#### Flow cytometry for C3b deposition assay

Bacterial C3b deposition was analyzed by flow cytometry. Briefly, *S. aureus* strains were grown to mid-log phase, washed twice with PBS, and resuspended. Bacterial suspensions were then incubated with an equal volume of normal human serum at 37°C for 30 minutes. After incubation, bacteria were washed and stained with a FITC-conjugated mouse anti-human C3b antibody (846108; Biolegend) in the dark at 4°C for 30 minutes. Following two washes, the bacteria were resuspended in PBS and analyzed on a flow cytometer. Data from at least 100,000 bacterial events per sample were collected. The level of C3b deposition was quantified as the median fluorescence intensity (MFI) in the FITC channel. Each experiment included 3 technical replicates and was performed with a minimum of 6 independent biological replicates; analysis was performed in a blinded manner.

#### Whole-blood killing assay

Bacteria were washed twice in PBS, diluted to an inoculum of 10^8^ CFU in 200 μL phenol red-free RPMI 1640, and mixed with 90 μL of freshly drawn human blood in 1.5 mL centrifuge tubes. Following a 2h incubation period at 37°C, cells were lysed using 0.01% Triton, at which time dilutions were plated on TSA to count the amount of CFU that was still alive. A one-way ANOVA was used to assess the statistical significance of the bacterial survival, with Tukey's multiple comparison test being used. This assay was performed with 5 independent biological replicates and 3 technical replicates per group; investigators were blinded to group allocation.

#### Neutrophil killing assay

The density gradient separation method was used to isolate healthy human neutrophils from whole blood as previously described.^50^ Erythrocytes were sedimented using dextran, lysed with ACK lysis buffer, and suspended in phenol red-free RPMI 1640 at the final concentration. In 1.5 mL centrifuge tubes, bacteria in the exponential growth stage were twice washed in PBS, diluted to 10^7^ CFU/ml, and then opsonized with 10% serum for 30 minutes. Neutrophils were challenged with *S. aureus* at a multiplicity of infection (MOI) of 10. Following a 30-minute incubation with mild agitation, cells were lysed in 0.2% Triton X-100 (Sigma-Aldrich), serially diluted in PBS, and plated on TSA for bacterial quantification. Statistical analysis was performed via one-way ANOVA with Tukey’s post hoc test to determine differences in survival rates across experimental groups. This assay was performed with 5 independent biological replicates and 3 technical replicates per group.

#### Cell culture and RAW 264.7 macrophage infection assay

According to earlier reports.^32^ Murine RAW 264.7 macrophages (1×10^6^ cells/mL) were maintained in serum-free DMEM (Gibco) within 48-well plates (0.5 mL/well), undergoing 6-hour pre-activation with IFN-γ (20 ng/mL) at 37°C. *S. aureus* cultures (mid-exponential phase, OD_600_=0.6) were PBS-washed and co-incubated with macrophages at MOI 10:1 (37°C, 30 min) to enable phagocytic uptake. Extracellular bacteria were eradicated by 1-hour gentamicin treatment (200 μg/mL), followed by dual PBS rinses. Initial bacterial loads (t=0) were quantified post-lysis (0.02% Triton X-100) via dilution plating. For longitudinal analysis, sustained low-dose gentamicin (12 μg/mL) exposure was maintained during subsequent 2-, 4-, and 24-hour incubations prior to terminal lysis. Five biological replicates with 3 technical replicates were analyzed for statistical robustness; investigators were blinded to group allocation during CFU counting.

#### Fluorescence microscopy

RAW 264.7 cells were cultured on glass coverslips for 2 hours in 24-well plates and then infected with WT_gfp and Δ*qorA*_gfp as previously outlined. Using 4% paraformaldehyde in PBS, the infected cells were fixed for 10 min and then stained for 30 min with Actin-Tracker Red-Rhodamine (Beyotime, C2207S). For 20 minutes. Hoechst dye was used to stain the nuclei. All samples were mounted with Prolong Diamond Antifade (Molecular Probes) and photographed using an Olympus FV3000 confocal microscope. Images were processed using Image J. Three slides (six images per slide) were analyzed per condition.

#### Mouse rapid blood clearance model

To investigate the role of *qorA* in *S. aureus* virulence, we used a rapid blood clearance model using 12 female outbred BALB/c mice (6–7 weeks old; weight 18–22 g; Beijing Vital River Laboratory Animal Technology Co., Ltd., (Certificate No. SCXK-2023-0012). Prior to experiment, mice were acclimatized for 7 days in a particular pathogen-free conditions with 50% humidity and 12 h of light/dark cycle. Animals were randomly allocated to three groups (*n*=4 mice/group) by block randomization (computer-generated sequence): (1) WT, (2) Δ*qorA* mutant, and (3) Δ*qorA* complemented with pOS1_*qorA*.

Mice received 1×10^8^ CFU of respective strains via tail vein injection (200 μL PBS), performed between 9:00–11:00 AM to minimize circadian effects. Blood samples (20 μL) were collected from the medial canthal vein at 0, 2, 5, 10, 20, and 30 min post-infection. At 30 min, mice were euthanized by intraperitoneal injection of tribromoethanol, followed by organ homogenization (heart, liver, spleen, lungs, kidneys) and bacterial enumeration on TSA plates. Inclusion required successful initial infection (blood CFU confirmation at t=0); no animals met exclusion criteria (predefined: injection-site inflammation or aberrant baseline counts).

During organ processing and the measurement of bacteria,experimenters were blinded to the identity of each group. Cage locations were changed every day to control the environmental confounders. Data are shown as mean log CFU/ml SEM. This animal experiment was independently repeated 3 times.

#### Mouse long-term bloodstream infection model

To assess bacterial colonization and survival during established infection, a long-term bloodstream infection model was employed. Groups of female outbred BALB/c mice were inoculated via tail vein injection with approximately 1×10^7^ CFU of the WT, Δ*qorA*, or pOS1-*qorA* strain (*n*=5 mice per group) in 100 μL PBS. At 24 hours post-infection, mice were euthanized. The heart, liver, spleen, lungs, and kidneys were aseptically collected, homogenized in PBS, and plated on TSA for bacterial enumeration. Results are expressed as mean log_10_ CFU per gram of tissue ± SEM. Investigators were blinded to group allocation during organ processing and CFU counting; this experiment was independently repeated 3 times.

#### Mouse *in vivo* competition model

For the competitive infection assay, the WT and Δ*qorA* strains were mixed in a 1:1 ratio (based on OD_600_) and inoculated into mice via tail vein injection as previously described. After 24 hours, target organs were collected and homogenized. Homogenates were plated on TSA to obtain isolated colonies. Subsequently, random colonies were genotyped by PCR amplification of the *qorA* locus to determine the proportion of each strain. This assay used *n*=5 mice per group, was performed with 3 independent biological replicates, and all procedures were conducted in a blinded manner.

#### Immunofluorescence double staining

Mouse organ tissues were collected from 5 mice post competitive bloodstream infection, fixed in 4% paraformaldehyde for 24 hours, embedded in paraffin, and serially sectioned (5 μm). After deparaffinization to water, antigen retrieval was performed with citrate buffer (pH 6.0) for 15 minutes, followed by cooling to room temperature. Permeabilization was conducted with 0.3% Triton X-100 for 10 minutes, and blocking with 5% BSA for 30 minutes. Primary antibody mixture (anti-Ly6G, GB11229, Servicebio; anti-F4/80, ab6640, Abcam) was added and incubated overnight at 4°C. After rewarming the next day, fluorescently labeled secondary antibody was added and incubated for 1 hour at room temperature in the dark. Nuclei were stained with DAPI for 5 minutes, and sections were mounted with anti-fluorescence quenching agent. Images were observed under a fluorescence microscope; fluorescence intensity was analyzed using ImageJ software. Three tissue sections were analyzed per mouse, with 3 technical replicates per section.

#### Mouse model of subcutaneous abscesses

For mouse model development, we used outbred immunocompetent female BALB/c mice (6 to 7 weeks old). Hair on the backs of these mice was removed using an animal shaver and depilation cream. For mouse infection, *S. aureus* cells were cultured in TSB medium to the mid-exponential phase of growth (OD_600_ = 0.6). The mice were anesthetized and inoculated with either 100 μL PBS or PBS containing ∼10^8^ live *S. aureus* via subcutaneous injection in the back. The bacterial burden in homogenized biopsy specimens was measured via serial dilution and plating on blood agar plates. This model used *n*=5 mice per group; investigators were blinded to group allocation during sample processing and CFU counting, and the experiment was independently repeated 3 times.

### Quantification and statistical analysis

All statistical analyses were performed using GraphPad Prism 8 software. Data are presented as mean ± standard error of the mean (SEM). Sample sizes (n) represent biological replicates as indicated in figure legends. *Student’s* t-test was used for two-group comparisons; one-way ANOVA followed by Tukey’s post hoc test was used for multiple-group comparisons. Statistical significance was defined as ∗*P <* 0.05, ∗∗*P <* 0.01, ∗∗∗*P <* 0.001, ∗∗∗∗*P <* 0.0001. Exact n values, statistical tests, and definitions of center (mean) and dispersion (SEM) are provided in all figure legends. Individual data points are plotted for all bar charts and boxplots. Asterisks represent significance levels and are defined in each figure legend.

### Additional resources

No additional resources, custom software, or novel tools were generated or used beyond the materials and methods described in this study.

## Supplemental information

Document S1. Figures S1–S3 and Table S1

Table S2. Transcriptomic data for the four clones

Table S3. Transcriptomic data for the WT and ΔqorA strains

Table S4. Metabolomic data for the WT and ΔqorA strains
