# Supplementary material for: qorA shapes organ-specific adaptation of ST59-MRSA via balancing immune evasion and metabolic trade-off
Source: iScience. 2026 Jun 11;29(7):116272. doi: 10.1016/j.isci.2026.116272 (PMC13276157; doi:10.1016/j.isci.2026.116272)
Supplement: Document S1. Figures S1–S3 and Table S1 [file mmc1.pdf]

iScience, Volume *29*

Supplemental information

qorA shapes organ-specific adaptation of ST59-MRSA via balancing immune evasion and metabolic trade-off

Fengning Chen, Yuyao Yin, Si Liu, Hongbin Chen, Yuzhen Wang, Bingqing Li, Yan Jin, and Hui Wang

Figure S1. Phagocytosis and intracellular survival of *S. aureus* in THP-1 cells.

I) The percentage of phagocytosed *S. aureus* in THP-1 cells. II) Percentage of *S. aureus* survival in activated THP-1 macrophages at 2 h. Related to Figure 4. Each experiment was repeated independently three times with at least four technical replicates per sample. Comparisons were made using one-way ANOVA with Tukey's post hoc test for multiple comparisons; Data are shown as mean ± SEM. **, $P < 0.01$; ****, $P < 0.0001$.

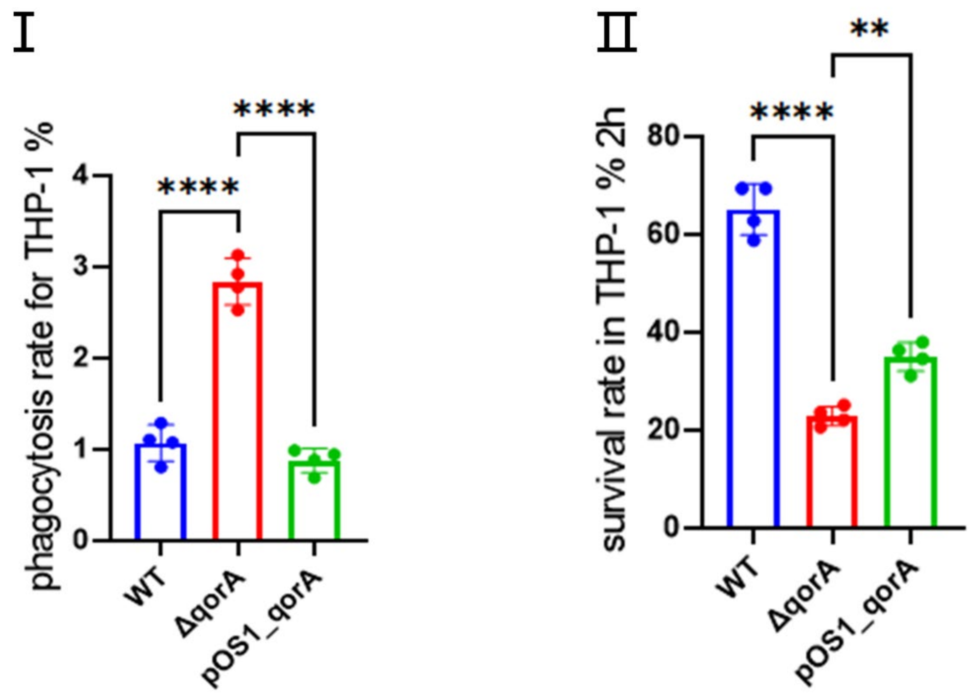

Figure S2. Effect of NAC pretreatment on phagocytosis of *S. aureus* strains by RAW264.7 cells. RAW264.7 cells were pre-treated with 0, 0.5 mM and 10 mM of NAC for 3 h, followed by comparison of the phagocytosis rates of WT, Δ*qorA* or the pOS1_*qorA* strains. Related to Figure 4. Each experiment was repeated independently three times with at least three technical replicates per sample. Comparisons were made using one-way ANOVA with Tukey's post hoc test for multiple comparisons; Data are shown as mean ± SEM. *, $P < 0.05$; ***, $P < 0.001$.

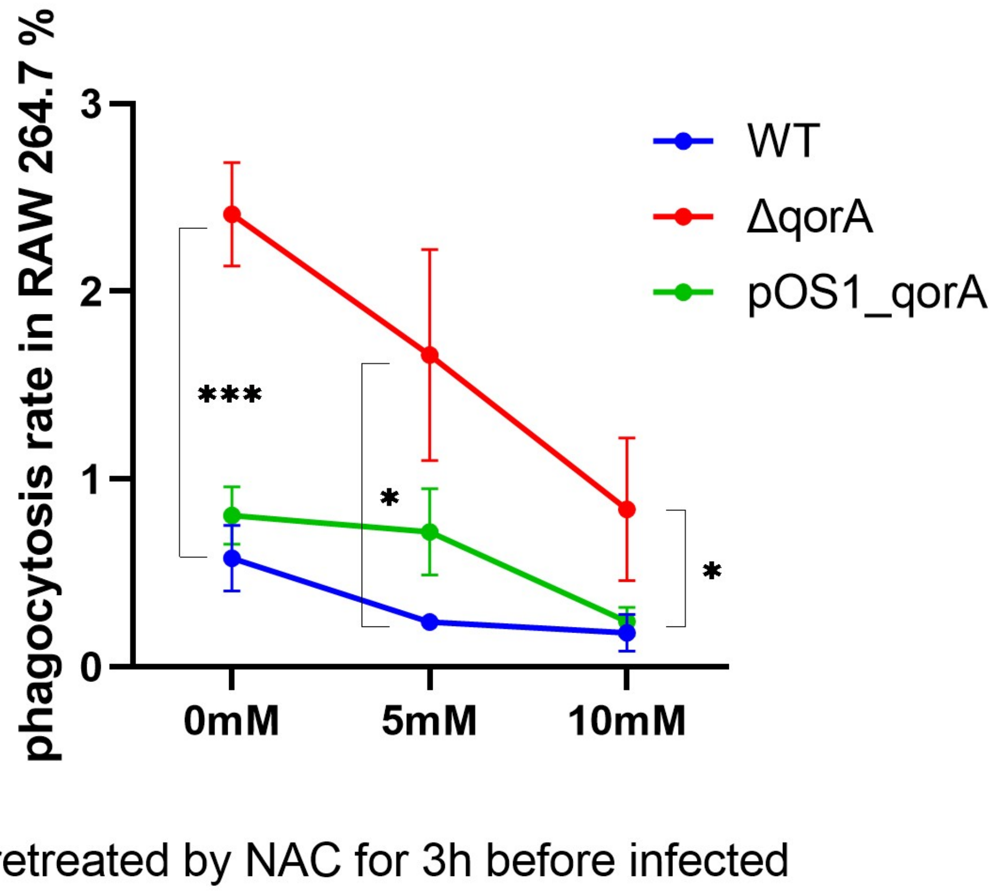

Figure S3. Representative immunofluorescence images of infected tissues.

Ly6G-positive neutrophils (green fluorescence), F4/80-positive macrophages (red fluorescence); cell nuclei stained with DAPI (blue fluorescence). Related to Figure 5.

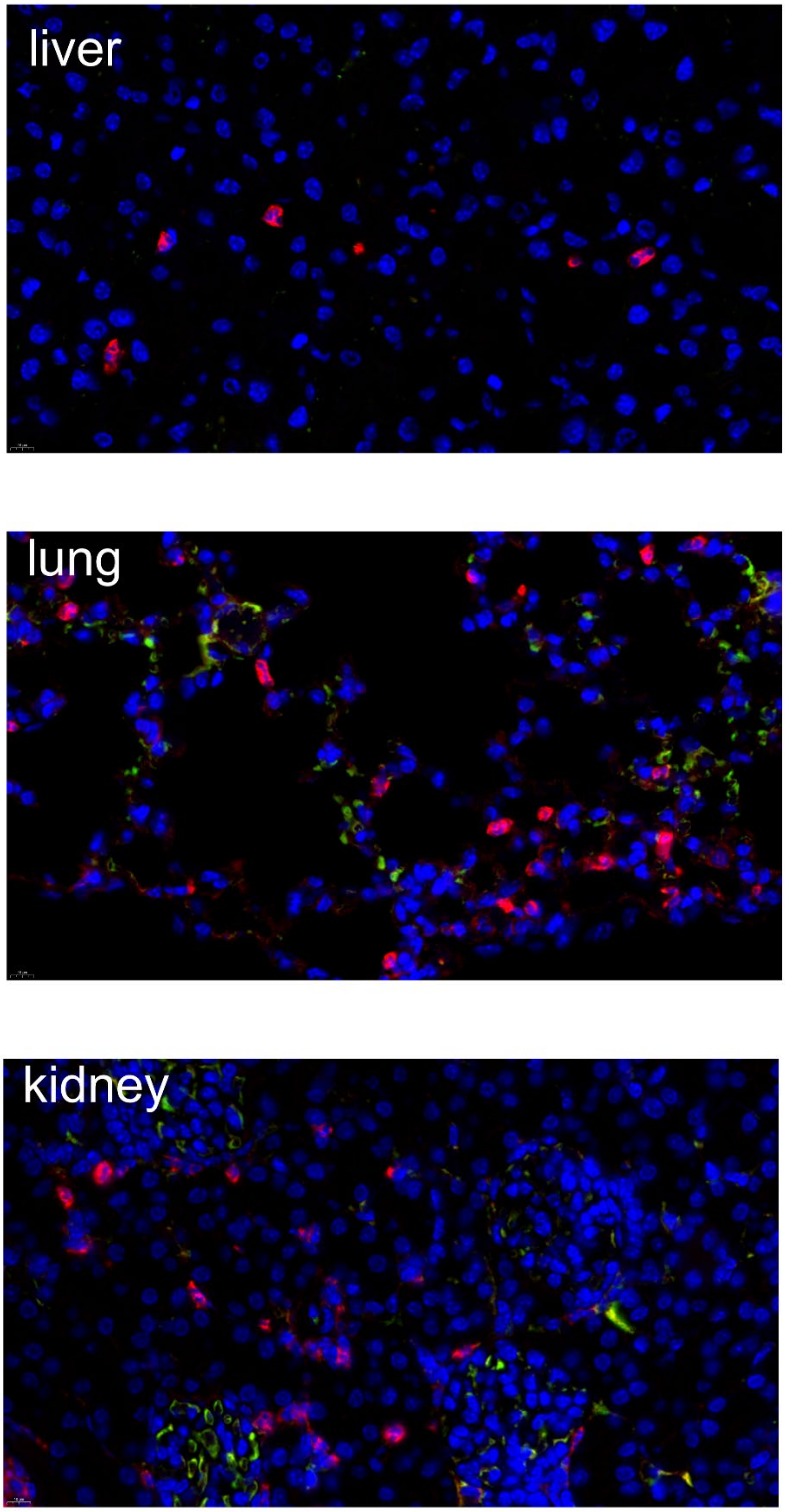

Table S1. Strains and primers used in this study. Related to STAR Methods.

**Strain**		
WT	A clinically isolated ST59-MRSA strain	
Δ*qorA*	A *qorA* gene knockout strain of the ST59 MRSA strain	
pOS1_*qorA*	A complemented strain of the Δ*qorA* strain	
WT_gfp	The ST59-MRSA strain transfected with the gfp fluorescent plasmid	
Δ*qorA* _gfp	The Δ*qorA* strain transfected with the gfp fluorescent plasmid	
**Primer**	**Sequence (5'→3')**	**Purpose**
qorA _F_q	AGCTGCTTCTGGTGCTGTAG	qRT-PCR
qorA _R_q	CGTCAAAGCCCAGTGTCTCT	qRT-PCR
qorA _F1_new	GATTTCATACACGGTGCCTGAGATGCAGTGAATCCTATTGT	Construct the Δ*qorA* strain
qorA _R1_new	CTTGTTTACCAAAGTTATCGCCGCATGCCTTCTGGTACTTTA	Construct the Δ*qorA* strain
qorA _F2_new	GGCGATAACTTTGGTAAACAAG	Construct the Δ*qorA* strain
qorA _R2_new	ATGCCTCAAGCTAGAGAGTCGTTGTTTCTTGTCGTGATGC	Construct the Δ*qorA* strain
pBT_sq_F	CCACCTGACGTCTAAGAAAC	Construct the Δ*qorA* strain
pBT_sq_R	GATGGTAACTTCACGGTAACGAT	Construct the Δ*qorA* strain
qorA _conf_F_long	CATGGATTTGGTGGTAGTGCC	Construct the Δ*qorA* strain
qorA _conf_R_long	CGCGCAAACATAAATTCATGTG	Construct the Δ*qorA* strain
qorA _400_F	AGAGACACTGGGCTTTGACG	Construct the pOS1_*qorA* strain
qorA _400_R	TTTTAAATGCACGTGGCGCA	Construct the pOS1_*qorA* strain
qorA _com_F	TTACGCCAAGCTAGCTTGGCTGCAGGCAACTGCTAGTTTTCTGGG	Construct the pOS1_*qorA* strain
qorA _com_R_short	GCCTTAAAGACGATCCGGGGAATTCAACAAAGTTGGTTTGTTAAAACG	Construct the pOS1_*qorA* strain
POS1_F	CTGCAGCCAAGCTAGCTTGG	Construct the pOS1_*qorA* strain
POS1_R	GAATTCCCCGGATCGTCTTT	Construct the pOS1_*qorA* strain
